# Purification and Nitrogen Doping of Nanothin Exfoliated Graphite Through RF Thermal Plasma Treatment

**DOI:** 10.3390/nano9070995

**Published:** 2019-07-10

**Authors:** Byung-Koo Son, Kyu-Hang Lee, Tae-Hee Kim, Myung-Sun Shin, Sun-Yong Choi, Guangsup Cho

**Affiliations:** 1 Department of Electrical and Biological Physics, Kwangwoon University, Seoul 01897, Korea; 2 Division of Plasma Convergence R&D, Cheorwon Plasma Research Institute, Cheorwon 24047, Korea; 3 Institute for Nuclear Science and Technology, Jeju National University, Jeju 63243, Korea

**Keywords:** nitrogen-doped graphite, N doping, nanothin exfoliated graphite, RF thermal plasma, purification

## Abstract

A mixture of nanothin exfoliated (NTE) graphite and urea (CO(NH_2_)_2_) powder was treated with radio frequency (RF) thermal plasma to achieve in situ purification and nitrogen doping of NTE graphite using the high-temperature flame of the RF plasma. Reactive species such as NH_3_, NH_2_, and HCNO generated by the thermolysis of urea play an important role in the purification and nitrogen doping of NTE graphite. The nitrogen content of NTE graphite subjected to plasma treatment increased by 5 times compared with that of raw NTE graphite. Three types of nitrogen species, namely, quaternary N, pyridinic N, and pyrrolic N, were observed after N doping with plasma treatment. The sheet resistance of N-doped NTE graphite reduced to 12–21% compared to that of the untreated NTE graphite, with the corresponding resistivity being ~7 × 10^−6^ Ω m.

## 1. Introduction

Since the discovery of the excellent physical properties of graphene, numerous studies have been carried out to apply graphene in industry [1,2,3]. Graphene is produced by two approaches: (1) chemical vapor deposition (CVD) of graphene is performed for electronic and barrier applications [4,5,6], and (2) nanothin exfoliated graphene flakes (NTE graphite) are obtained by compounding graphene with polymers for developing composite materials [7,8,9,10]. Although the physical properties of NTE graphite are not equivalent to those of graphene, NTE graphite shows preferable performance because it is thinner than conventional composite graphite compounded with a reinforced polymer for enhancing thermal and electrical conductivity and mechanical properties. Since graphene has a charge mobility of 200,000 cm^2^/V at room temperature, it has high electrical conductivity even when combined with conductive polymers, but its mobility also depends on the transfer method and the substrate used [11]. However, it is not easy to enhance the electric conductivity of graphite, owing to the high contact resistivity that disrupts electron movement. Because NTE is almost as thin as graphene, it can form conductive channels when compounded with a polymer, giving the composite improved conductivity.

NTE graphite is conventionally synthesized by wet chemical methods through oxidation and reduction processes [12,13,14]. There are two different methods for producing NTE graphite by oxidation: (1) the Brodie and Staudenmaier method and (2) Hummers’ method. In the Brodie and Staudenmaier method, nitric acid (HNO_3_) combined with potassium chlorate (KClO_3_) is used as the oxidant to prepare graphite oxide (GO). Meanwhile, in Hummers’ method, potassium permanganate (KMnO_4_) combined with sulfuric acid (H_2_SO_4_) is used as the oxidant. Currently, Hummers’ method is the most commonly used technique for producing NTE graphite. Further, NTE graphite is obtained by the reduction of GO powder using hydrazine, dimethylhydrazine, hydroquinone, or NaBH_4_ as reducing agents [15,16,17].

The physical properties of NTE graphite produced by the above chemical processes are quite different from those of graphene. In order to improve the electrical properties of NTE graphite composite compounds, the quality of the NTE graphite should be improved; this is because NTE graphite processed by chemical methods contains various crystal defects and imperfections at a higher density than does natural graphite.

The objective of this work is to improve the electrical performance of NTE graphite. Its purification and modification were carried out during the nitrogen doping process for the production of NTE graphite powder using radio frequency (RF) thermal plasma. Although there are numerous studies on the surface modification and doping of CVD-grown graphene, very few groups have reported the production of NTE graphite powder because of the lack of effective processing technologies [17,18]. The RF thermal plasma process has been widely used for the production of nanomaterials owing to its unique thermal characteristics [19,20,21,22]. Thermal plasma offers a high-temperature medium with temperature over 10,000 K, and the temperature gradient is very sharp with a high flow velocity of a few hundred meters/second. The raw material (e.g., generally in the powder form) is fed into the hot zone created by the plasma, and the residence time of the powder passing through this hot zone is typically ~1–3 ms. Most of the injected powder is vaporized at the high temperature of the plasma flame; however, we selectively controlled the vaporization and functionalization of the powders by controlling the operating and process conditions. A relatively low plasma input power of 30 kW and the vertical thermal conductivity of the NTE graphite allow the purification and modification of the NTE graphite powder. In addition, the improvement in the properties of NTE graphite produced by the thermal plasma doping process was evaluated by measuring the sheet resistance of purified N-doped NTE graphite.

## 2. Experimental Details

### 2.1. Materials

For the N doping process, NTE graphite powder (H-15, XG Sciences, Inc., East Lansing, MI, USA) and urea (CO(NH_2_)_2_) (Dongbu Urea, Farm Hannong Co., Ltd., Seoul, Korea) were used as the raw materials. The NTE graphite has an average thickness of ~15 nm, typical surface area of 50–80 m^2^/g, and particle diameter of ~15 μm. The commercial sample originally contained sulfur and chlorine as impurities, which were expected to be degraded during the exfoliation process of the fabrication. Urea is completely vaporized due to its low melting point (132.7 °C) and boiling point (165.1 °C), while highly heat-resistant NTE graphite suffers no deformation at the high temperature of the plasma flame. For effective nitrogen doping of NTE graphite, the urea powder was first dissolved in deionized water; then the NTE graphite powder was mixed with the aqueous solution of urea and the mixture was dried in an oven. The so-obtained NTE graphite and urea mixture in powder form was used as the raw material.

### 2.2. Configuration of the RF Plasma System

**Figure 1:**
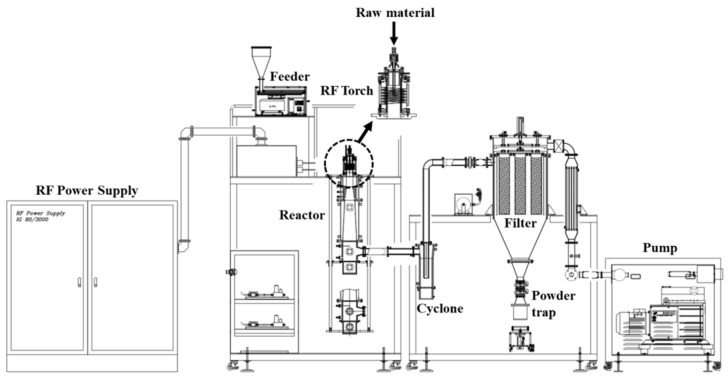
Schematic of the RF thermal plasma system.

Figure 1 shows a schematic of the RF thermal plasma torch system developed in our laboratory [23]. The system consists of an RF power supply (TruHeat HF7080, TRUMPF Inc., Ditzingen, Germany), torch, powder feeder, reactor, cyclone, and collector with filter. A copper induction coil with Teflon insulation was used, and the confinement tube inside the torch was made of silicon nitride to prevent thermal shock. The discharge gas for generating the plasma flame was supplied by two routes as central and sheath gases. The central gas was injected through various holes to form a swirl pattern for achieving a homogeneous supply of the discharge gas, and the sheath gas was vertically injected from the rim of the confinement tube. The central gas directly generates the plasma flame. The sheath gas protects the confinement tube from thermal damage and concentrates and confines the heat to the plasma by preventing the thermal adsorption of evaporated particles. The raw materials were loaded into the plasma reactor using a vibrating powder feeder. The synthesized products were collected at the collector shown in Figure 1. Sintered metal filters made of porous stainless steel with 500 nm pore size were installed at the collector. The powder attached to the surface of the filters was blown away by the compressed gas and collected in the powder trap underneath the filter. The reactor pressure was controlled by a rotary pump. The one-step synthesis process progressed in the order of gas injection, RF plasma discharge, supply of the raw material, N doping, and filtering.

### 2.3. Synthesis of Nitrogen-Doped NTE Graphite

The experimental specifications are listed in Table 1. The input power for RF plasma generation was controlled to a relatively low value of ~30 kW to prevent the vaporization of the NTE graphite powder, which has low thermal conductivity in the vertical direction, and to selectively decompose the urea powder only. Argon was used as the plasma forming gas; it was flown into the plasma torch as the central and sheath gases at 30 and 50 L/min, respectively. The experiment was carried out at 350 Torr pressure in the reactor chamber. The powder NTE graphite and urea mixture was fed into the hot zone of the thermal plasma from the injector at the center of the RF torch, as shown in Figure 1, at a feeding rate of 1 kg/h with an Ar carrier gas flow rate of 10 L/min. The synthesis was performed for 1 h, and ultimately, the produced powder was collected at the rate of ~950 g/h at the inner wall of the system.

The characteristics of N-doped NTE graphite synthesized by plasma treatment were compared with those of untreated NTE graphite. The morphological deformation of the plasma-treated product was observed by field-emission scanning electron microscopy (FE-SEM; S-4800, Hitachi, Tokyo, Japan) at an accelerating voltage of 5 kV. The atomic composition was analyzed using an Organic Element Analyzer (OEA; Flash 2000, Thermo Fisher Scientific, Waltham, MA, USA) and an X-ray photoelectron spectrometer (XPS; ESCA Sigma Probe Spectrometer, Thermo Fisher Scientific, Loughborough, UK). The crystalline structure was analyzed by X-ray diffraction (XRD; D2 PHASER, BRUKER Co., Berlin, Germany) with Cu Kα radiation. The structural imperfection was examined by Raman spectroscopy (Senterra, Bruker, Karlsruhe, Germany) with a 785 nm laser at the power of 100 mW. In order to investigate the electrical properties of NTE graphite, the powders of untreated and N-doped NTE graphite were pressed into graphite sheets and the surface resistance of the sheets was measured.

## 3. Results and Discussion

### 3.1. Analysis of N-Doped NTE Graphite and Untreated NTE Graphite

**Figure 2:**
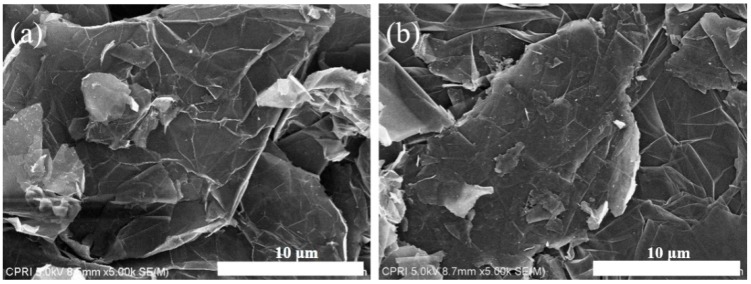
SEM images of (**a**) untreated NTE graphite and (**b**) N-doped NTE graphite.

Figure 2a,b shows the FE-SEM images of the NTE graphite powder before and after plasma treatment, respectively. The images reveal that the surface morphology of NTE graphite did not change after the N doping process using the RF thermal plasma, which suggests that NTE graphite did not deform at the high temperature in the reaction chamber, where only the urea vaporized and reacted with NTE graphite in the RF plasma.

**Figure 3:**
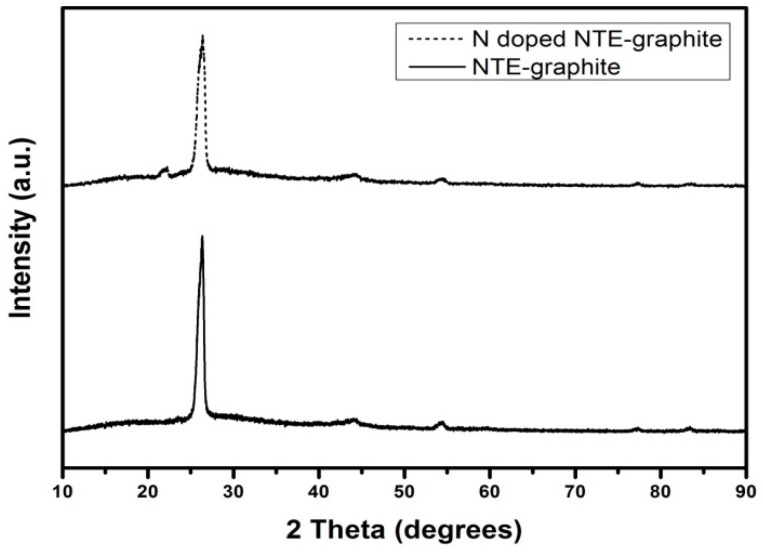
XRD patterns of untreated NTE graphite (solid line) and N-doped NTE graphite (dotted line).

Figure 3 shows the XRD patterns of raw and N-doped NTE graphite. In both patterns, the (002) crystalline plane is observed at 2*θ* = 26.3°. The XRD pattern of N-doped NTE graphite shows a new peak at 2*θ* = 22°, which corresponds with the (002) plane of the graphitic interlayer spacing for N-doped graphene [24]. The appearance of this new peak suggests that some NTE graphite units were partially exfoliated during the thermal plasma treatment. Further, it reveals that NTE graphite and urea were mixed well in deionized water, and oxidation of a part of the graphite could have occurred. During the plasma heating process, gas molecules (oxygen, nitride, hydrogen, and other gaseous compounds) are generated and dissolved as reactive species in the thermal plasma. The reactive molecules contribute to N doping, and exfoliation with the evaporated gases leads to expansion of the inter layers of graphite. Thus, the exfoliation of graphite occurs at the excessive thermal pressure which aids in overcoming the van der Waals interlayer attraction between the graphite layers [25].

In addition, the thickness of NTE graphite crystallites was estimated by calculating the crystallite size (*τ*) using the Scherrer equation, *τ* = *Kλ*/*β*cos *θ*, where *K* is the shape factor, *λ* is the wavelength of the X-ray (1.54184 Å), *β* is the full width at half-maximum, and *θ* is the Bragg angle. The crystallite size of untreated NTE graphite was calculated to be 104.5 Å, while that of N-doped NTE graphite was calculated to be smaller at 85.4 Å. The decrease in the crystallite size can be attributed to the enhancement of imperfections due to the generation of crystal defects during nitrogen incorporation in the N doping process.

**Figure 4:**
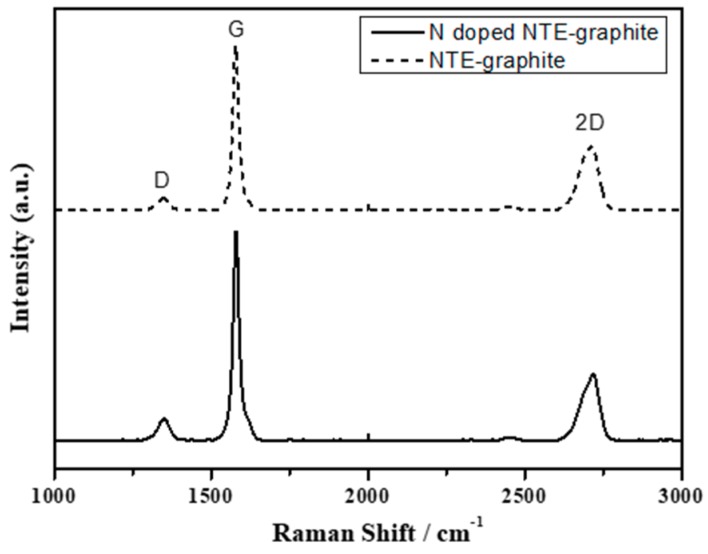
Raman spectra of untreated NTE graphite (dotted line) and N-doped NTE graphite (solid line).

Figure 4 shows the Raman spectra of untreated NTE graphite and plasma-treated N-doped NTE graphite. In both spectra, D, G, and 2D bands are observed at 1345, 1580, and 2710 cm^−1^, respectively. The disorder peak (D-band) is due to the vibration mode of A_1g_ owing to the structural defects and other disordered structures in the graphitic plane, while the graphitic peak (G-band) corresponds to the E_2g_ vibration mode of C–C bond stretching in the carbon structure with sp2 carbons. The intensity ratio (*I*_D_/*I*_G_) of the D and G bands was compared to investigate the change in the structural defects after the N doping process. The *I*_D_/*I*_G_ ratio of NTE graphite increased from 0.075 to 0.103 after the plasma treatment, revealing an increase in the number of defects in N-doped NTE graphite due to the incorporation of nitrogen atoms into the graphite layers.

Through elemental analysis, the amount of nitrogen doped and the purification of NTE graphene, i.e., the removal of sulfur impurities from raw NTE graphite, were estimated (see Table 2). The carbon content was above 90 wt % in NTE graphite before and after plasma treatment, while the nitrogen content increased fivefold from 0.83 to 3.99 wt % after N doping by the plasma process, indicating effective N doping. Moreover, the sulfur impurities were perfectly removed as they were not detected in N-doped NTE graphite. The contents of other elements, oxygen and unknown elements, decreased from 6.56 wt % for untreated NTE graphite to 4.68 wt % for N-doped NTE graphite.

**Figure 5:**
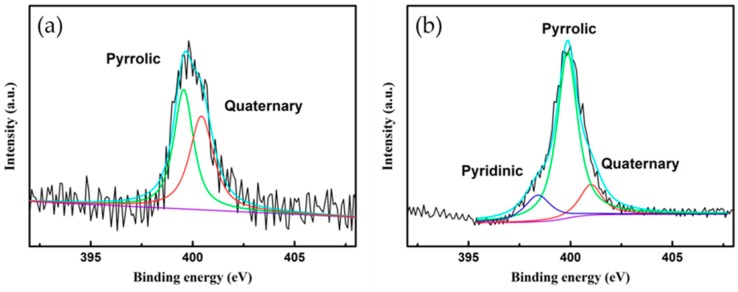
XPS spectra of (**a**) untreated and (**b**) N-doped NTE graphite. The deconvolution of the peak results in three subpeaks. Red line: Quaternary N, green line: Pyrrolic N, violet line: Pyridinic N, and greenish-blue line: the sum of the deconvoluted spectrum.

Figure 5 shows the XPS spectra of untreated NTE graphite (a) and N-doped NTE graphite (b). Typically, N-doped graphene has three kinds of nitrogen bonding structure in the form of quaternary N (or graphitic N), pyridinic N, and pyrrolic N. The pyridinic N is bonded with two carbon atoms that are bound to nitrogen at the edge. It contributes to a *p*-orbital electron in the π-electron system. Pyrrolic N dedicates two *p*-orbital electrons to the π-electron system. Quaternary N is formed by the substitution of a C atom with an N atom. In the deconvoluted XPS spectrum of NTE graphite (Figure 5a), the peaks of pyrrolic N and quaternary N are observed at the binding energies of 399.6 and 400.4 eV, respectively. In the deconvoluted XPS spectrum of N-doped NTE graphite (Figure 5b), those peaks are observed at 399.8 and 401 eV, respectively, along with an additional peak of pyridinic N at 398.4 eV [26,27,28].

Table 3 shows the atomic ratios of nitrogen atoms with different bonding structures calculated using the XPS data. As shown above in Figure 5, pyrrolic bonding formed at 0.26 atom % after N doping, while the atomic compositions of pyridinic and quaternary nitrogen improved by 7 and 1.5 times, respectively. The total atomic percent of nitrogen bonded with carbon in NTE graphite increased fivefold from 0.36 to 1.79 atom % after the N doping process. The results of XPS analysis agree well the elemental composition determined by elemental analysis after N doping, shown in Table 2.

### 3.2. Electrical Performance of the N-Doped NTE Graphite Sheet

In order to evaluate the improvement in the physical properties of NTE graphite after the N doping process, the surface resistance (*R*_s_) of sheets of untreated and N-doped NTE graphite was measured (Table 4). The NTE graphite and N-doped NTE graphite were pressed into sheets by the hot press method after supersonic treatment and vacuum filtration of the graphite. The resistance of the two so-formed sheets was measured and then re-measured again after a roll press process. The roll-pressed sheets showed improved electric conductivity compared to the sheets processed only by hot pressing. The sheet resistance was measured at eight positions, and the values are listed in the upper part of Table 4; the average value of these was calculated, as listed with the other parameters of the material such as the thickness, calibrated resistance, and resistivity in the lower part of Table 4. As the thickness of the sheet was varied, *R*_s_ was calibrated with the base thickness value of 100 μm. Without roll press processing, the resistance of N-doped NTE graphite was found to be ~12% lower (76.2 mΩ/sq) than that of untreated NTE graphite (86.4 mΩ/sq). After roll pressing, the sheet resistance decreased by ~21 % from 86.07 to 67.76 mΩ/sq due to N doping. The untreated NTE graphite sheet had almost the same thickness and resistance after roll pressing. However, the thickness and resistance of the N-doped NTE graphite sheet decreased by ~10% after roll pressing. The resistivity of N-doped NTE graphite was calculated to be ~7 × 10^−6^ Ω m, which is 10 times lower than that of CVD-grown graphene [29,30]. The electrical performance of NTE graphite, that is, its sheet resistance, improved after the N doping process, and the interfacial resistivity between each NTE graphite flake improved owing to the separation of graphite during the thermal plasma treatment.

### 3.3. N Doping Process in the RF Thermal Plasma System

**Figure 6:**
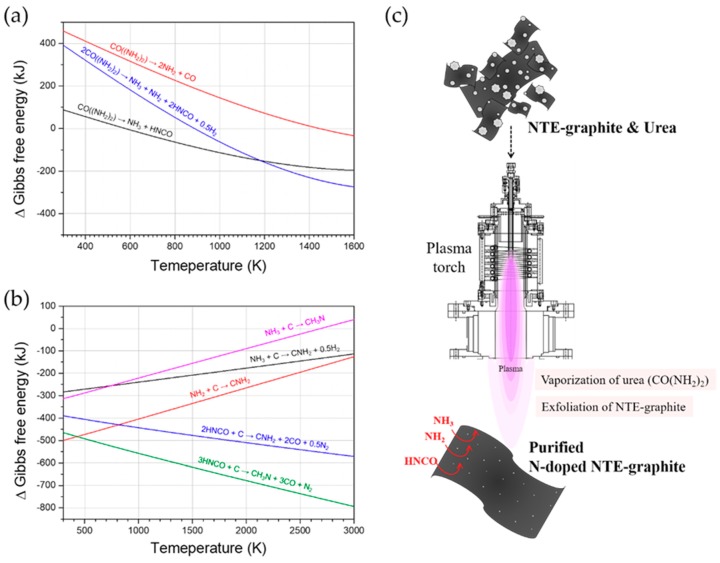
The variation in the Gibbs free energy: (**a**) The decomposition of urea and (**b**) the generation of C_x_N_y_H_z_ radicals. (**c**) An illustration of the N doping of NTE graphite in the RF thermal plasma system.

Urea is fully vaporized at the high temperatures ranging from 10,000–12,000 K in the plasma center of the plasma flame because it has low melting (132.7 °C) and boiling (165.1 °C) temperatures. The spontaneity of the decomposition of urea and the generation of reactive C_x_N_y_H_z_ radicals was theoretically verified using a simulation of the Gibbs free energy (HSC chemistry ver. 9.1.1, Qutotec Research Oy, Espoo, Finland) as depicted in Figure 6a,b. In general, urea decomposes and generates NH_3_ and ammonium salt molecules (RNH_2_), which react with NTE graphite during the N doping process [26,31]. In addition, according to the established mechanism, urea thermolyzes into NH_3_ and isocyanic acid (HNCO) [32]. Using the thermodynamic equilibrium calculation with the HSC chemistry software shown in Figure 6a, urea was predicted to decompose into NH_3_ and HNCO at temperatures over 580 K with a negative change in the Gibbs free energy. Further, NH_2_ radicals are generated at temperatures greater than 1450 K. All of the aforementioned species are generated above 890 K. These chemical reactions easily take place during the thermal plasma process because of the high temperature of the chamber, which is a few thousand degrees. As shown in Figure 6b, CNH_2_ and CH_3_N radicals are generated below the temperature of 3000 K on the surface of NTE graphite. This simulation revealed that NH_2_ and NH_3_ species and HCNO have high reactivity with NTE graphite. Although the sublimation temperature of graphite at atmospheric pressure lies between 3895 and 4020 K, graphite with its high thermal stability does not evaporate or melt in the high-temperature plasma flame; instead, it can react with the NH_3_, NH_2_, and HNCO species generated from urea.

The synthesis process of N-doped NTE graphite during thermal plasma processing is illustrated in Figure 6c. When the mixed powder of NTE graphite and urea is injected into the high-temperature region inside the torch, urea vaporizes and infiltrates the graphite powder. Some graphite layers are exfoliated because the high pressure of the expanding gases exceeds the van der Waals interaction forces of the interlayers. The active species (i.e., NH_2_, NH_3_, and HNCO) generated by the decomposition of urea react with the NTE graphite and C–N–H bonds are formed, leading to N doping. Consequently, N-doped NTE graphite and a small amount of N-doped graphene are produced, while the sulfur impurities are removed during the thermal plasma treatment, leading to the purification of graphite.

## 4. Conclusions

N-doped NTE graphite was synthesized from a mixed powder of NTE graphite and urea by an RF thermal plasma process. During this process, the sulfur impurities in the raw NTE graphite were also eliminated, while the morphology of the NTE graphite was maintained. The nitrogen content of the doped material increased fivefold compared to that of raw NTE graphite after plasma treatment, as determined by OEA and XPS analyses. The content of nitrogen increased from 0.59 to 3.99 wt %, as determined by OEA, and the bonding percentage of nitrogen improved from 0.36 to 1.79 atom % after the N doping process. The reduction in the crystallite size of NTE graphite determined by XRD analysis arises from the exfoliation of graphite during the process of N doping. Further, an increase in defects after the N doping process was deduced from the enhancement of the *I*_D_/*I*_G_ ratio in the Raman spectrum. For the measurement of the resistance, thin sheets of graphite were fabricated with ~100 nm thickness from the powders of raw NTE graphite and N-doped NTE graphite by hot pressing and roll pressing. The measured sheet resistance indicated an improvement in the electrical performance of N-doped NTE graphite. The hot-pressed sheet of N-doped NTE graphite showed a 12% reduction in resistivity (7.61 × 10^−6^ Ω m) when compared to an untreated NTE graphite sheet (8.63 × 10^−6^ Ω m) processed similarly. Further, the resistivity decreased by 21.5% for sheets subjected to an additional roll press process, from 8.63 × 10^−6^ to 6.77 × 10^−6^ Ω m, which is 10 times lower than the resistivity of CVD-grown graphene.

The reactive species are generated by the decomposition of urea into NH_3_ gas, NH_2_ radicals, and HNCO molecules according to the thermodynamic equilibrium calculation. The RF thermal plasma process is a promising technology for the mass production of NTE graphite in a short duration owing to the high-enthalpy environment. The rapid expansion of gases at the ultrahigh temperature of plasma leads to a fast plasma fluid which might effectively contribute to N doping. In this work, N-doped NTE graphite was produced with accompanying elimination of sulfur impurities in a single-step plasma process and without the GO synthesis step.

## Figures and Tables

**Table 1:** Experimental conditions for the purification and N doping of nanothin exfoliated (NTE) graphite.

Plate Power	Plasma Forming Gas		Raw Material	Feeding Rate of Raw Material	Carrier Gas
	Central Gas	Sheath Gas			
30 kW	30 L/min Ar	50 L/min Ar	Mixed powder ^1^ of NTE graphite and CO(NH_2_)_2_	1 kg/h	10 L/min Ar

^1^ NTE graphite and urea (CO(NH_2_)_2_) powders were mixed in water and dried in an oven.

**Table 2:** Elemental composition of untreated NTE graphite and N-doped NTE graphite.

Materials	Elements (wt%)			
	C	N	H	S
Untreated NTE graphite	91.73	0.83	0.29	0.59
N-doped NTE graphite	90.85	3.99	0.48	-

**Table 3:** X-ray photoelectron spectroscopy (XPS) atomic compositions of untreated NTE graphite and N-doped NTE graphite in atomic percent.

Materials	Bonding Structure			Total
	Pyrrolic	Pyridinic	Quaternary	
Untreated NTE graphite	0.18	-	0.18	0.36
N-doped NTE graphite	0.26	1.28	0.26	1.79

**Table 4:** Resistance values of the sheets derived from untreated NTE graphite and N-doped NTE graphite.

**Materials**	**Sheet Resistance (*R*_s_, mΩ/sq)**							
	**1**	**2**	**3**	**4**	**5**	**6**	**7**	**8**
**Untreated NTE Graphite** **+ Hot PRESS**	80.34	80.7	75.37	74.88	77.36	79.51	71.17	72.33
**N-doped NTE Graphite** **+ Hot PRESS**	58.63	62.56	65	74.48	67.34	62.23	71.68	64.48
**Untreated NTE Graphite** **+ Roll Process**	78.77	75.41	74.32	76.09	79.55	80.92	72.33	82.93
**N-doped NTE Graphite** **+ Roll Process**	58.21	62.97	59.88	63.73	63.73	64.98	68.36	62.43
**Materials**	**Average *R*_s_** **(mΩ/sq)**		**Thickness** **(μm)**		**Calibrated *R*_s_ (mΩ/sq at 100 μm)**		**Resistivity** **(Ω m)**	
**Untreated NTE Graphite** **+ Hot PRESS**	76.46		113		86.40		8.63 × 10^−6^	
**N-doped NTE Graphite** **+ Hot PRESS**	65.80		115.8		76.20		7.61 × 10^−6^	
**Untreated NTE Graphite** **+ Roll Process**	77.54		111		86.07		8.60 × 10^−6^	
**N-doped NTE Graphite** **+ Roll Process**	63.04		107.5		67.76		6.77 × 10^−6^	
